# Suspected Murine Typhus Associated With Neurologic Symptoms and Posterior Reversible Encephalopathy Syndrome (PRES)-Like MRI Abnormalities: A Case Report

**DOI:** 10.7759/cureus.111616

**Published:** 2026-06-27

**Authors:** Nicholas W Tyndall, Franchesca Farris-Cosme, William Colthorpe, Gregory D Bowling

**Affiliations:** 1 Internal Medicine, Brooke Army Medical Center, San Antonio, USA; 2 Internal Medicine, San Antonio Uniformed Services Health Education Consortium, San Antonio, USA; 3 Hospital Medicine, UT Health San Antonio, San Antonio, USA

**Keywords:** murine typhus, plasma donation, posterior reversible encephalopathy syndrome (pres), rickettsia, rickettsia typhus

## Abstract

Murine typhus, caused by *Rickettsia typhi*, is a flea-borne zoonosis that typically presents with fever, headache, myalgias, and rash. Central nervous system involvement is uncommon, and neuroimaging findings that mimic posterior reversible encephalopathy syndrome (PRES) are rarely reported. This case highlights a rare neuroinfectious presentation of murine typhus with PRES-like imaging findings in a young woman.

A previously healthy woman in her 30s presented with persistent retro-orbital headaches, intermittent fevers, tremors, dysarthria, photophobia, myalgias, progressive weakness, and recurrent syncopal episodes. Initial computed tomography of the head demonstrated age-advanced microvascular ischemic changes versus possible PRES. Brain magnetic resonance imaging later revealed bilateral parietal-predominant fluid-attenuated inversion recovery (FLAIR) hyperintensities in the white matter, concerning for PRES. Cerebrospinal fluid (CSF) analysis was unremarkable, and extensive infectious and autoimmune evaluation was unrevealing. Despite nondiagnostic testing, persistent fevers and neurologic symptoms prompted empiric doxycycline therapy for suspected atypical infection. Within 48 hours, the patient experienced marked clinical improvement with resolution of fever, dysarthria, tremors, photophobia, and severe headache. Post-discharge serology showed a positive *Rickettsia typhi* IgM with a negative IgG. While these findings were consistent with possible early murine typhus, the absence of convalescent serologic testing limited definitive confirmation.

Murine typhus is increasingly recognized as a cause of severe neurologic disease due to endothelial injury and systemic vasculitis. This case is notable for PRES-like neuroimaging abnormalities, normal CSF studies, and severe neurologic manifestations occurring in an otherwise healthy host without traditional risk factors for severe disease.

The patient’s rapid clinical improvement following doxycycline therapy was consistent with a rickettsial infection but was not diagnostic of murine typhus. This case highlights the importance of early empiric treatment when rickettsial disease is suspected while awaiting confirmatory testing. This case expands the recognized neurologic spectrum of murine typhus and emphasizes the importance of considering rickettsial infections in patients presenting with unexplained febrile neurologic syndromes and nonspecific MRI abnormalities, particularly in endemic regions.

## Introduction

Murine typhus, caused by *Rickettsia typhi*, is a flea-borne zoonosis transmitted primarily through infected rat fleas. The disease remains endemic in many subtropical and coastal regions, including parts of the southern United States, and is often underrecognized because its clinical manifestations overlap with other febrile illnesses [1]. Patients typically present after a one- to two-week incubation period with nonspecific symptoms, such as fever, headache, myalgia, and malaise, with some developing rash, gastrointestinal symptoms, or mild elevation in liver-associated enzymes. Although most cases follow a relatively benign course with appropriate antimicrobial therapy, atypical presentations can complicate diagnosis and delay treatment [2].

Central nervous system (CNS) involvement in murine typhus is rare and usually manifests as mild neurologic symptoms such as headache. More severe neurologic complications, including aseptic meningitis or meningoencephalitis, have been reported but occur in approximately 2% to 5% of cases [3]. Neuroimaging abnormalities are rarely described, and radiologic findings mimicking posterior reversible encephalopathy syndrome (PRES) are particularly unusual. PRES is typically associated with conditions such as hypertension, renal failure, cytotoxic medications, and systemic inflammatory states, and is characterized by vasogenic edema predominantly involving the parietal and occipital lobes [4].

This case highlights an atypical presentation of suspected murine typhus with neurologic manifestations in a previously healthy young woman and magnetic resonance imaging (MRI) findings concerning for possible PRES-like features. While there was no evidence of meningitis, encephalitis, or direct CNS infection, the case underscores the diagnostic challenges posed by nonspecific neurologic and systemic symptoms and emphasizes the importance of maintaining a broad infectious differential in patients presenting with unexplained febrile illnesses accompanied by neurologic symptoms. Early recognition and empiric treatment with appropriate antimicrobial therapy remain critical to minimizing morbidity and promoting favorable outcomes.

## Case presentation

A woman in her 30s, residing in Texas, with no significant past medical history and taking no daily medications, presented in the spring to the emergency department after approximately three days of progressive symptoms before her initial emergency evaluation. She reported persistent retro-orbital headaches and photophobia shortly after plasma donation, followed over the ensuing days by intermittent fevers up to 39 °C, lightheadedness, nausea, myalgias, chills, night sweats, tremors, dysarthria, early satiety, weight loss, transient dysuria, and two syncopal episodes occurring several days apart. The first syncopal episode occurred while donating plasma, during which she experienced sudden alternating sensations of heat and cold before losing consciousness. Before symptom onset, she had been in her usual state of health except for mild fatigue attributed to prior anemia. Given her constitutional symptoms, a broad differential diagnosis was considered, including infectious, inflammatory, and neoplastic etiologies. She denied recent travel, sick contacts, animal/outdoor exposures, lymphadenopathy, respiratory symptoms, gastrointestinal bleeding, or other localizing symptoms suggestive of an alternative primary process.

Before this presentation to the emergency department, the patient had also been evaluated at an outside hospital following her second syncopal episode. During this episode, she experienced symptoms similar to her first event, which occurred while walking in a grocery store. She was diagnosed with a migraine and treated with a migraine cocktail (1 liter of lactated Ringer’s, 2 g of magnesium sulfate, 15 mg of ketorolac, 25 mg of diphenhydramine, and 10 mg of metoclopramide), which provided temporary relief. Imaging was not completed at that time because the patient left to attend to childcare obligations.

The syncopal episodes occurred in the setting of an ongoing febrile illness and were preceded by prodromal sensations of heat and cold. One episode occurred during plasma donation. Although a definitive etiology was not established, the clinical features were initially felt to be most consistent with vasovagal or orthostatic syncope. Orthostatic vital signs could not be obtained due to the patient's unwillingness to stand secondary to weakness. Electrocardiography demonstrated a normal sinus rhythm without conduction abnormalities, and continuous telemetry during hospitalization revealed no arrhythmias. The patient appeared clinically dehydrated on exam. Hemoglobin and basic metabolic studies were unremarkable for a primary metabolic cause of syncope. Overall, the initial episode during plasma donation was most consistent with a vasovagal or volume-depletion event, whereas subsequent episodes were felt to be multifactorial in the setting of acute febrile illness and dehydration rather than cardiogenic syncope.

On presentation to the emergency department, her vital signs were notable for a blood pressure of 98/77 mmHg and a blood glucose level of 99 mg/dL. During hospitalization, vital signs demonstrated intermittent fevers (36.5-39.3 °C), heart rates of 59-116 bpm, and blood pressures ranging from 94-130 to 49-97 mmHg (mean arterial pressure (MAP) 62-105 mmHg), without sustained hypertensive episodes. Oxygen saturation remained 97-100% on room air. 

Neurologic examination revealed an awake, alert, and fully oriented patient. Attention was intact during conversation, and language function was normal, with preserved fluency, cadence, object naming, and phrase repetition without evidence of paraphasic errors or word-finding difficulty. Memory was intact, with appropriate recall of personal medical history and medications. Cranial nerve examination was unremarkable. Pupils were equal, round, and reactive to light bilaterally, visual fields were full, and extraocular movements were intact without nystagmus. Facial sensation and movements were symmetric, hearing was intact bilaterally, palate elevation was symmetric, shoulder shrug strength was preserved, and the tongue protruded in the midline. Funduscopic examination was limited because the optic discs could not be adequately visualized without pupillary dilation. No nuchal rigidity or meningeal signs were present, and Kernig and Brudzinski signs were negative.

Motor examination demonstrated normal muscle bulk and tone throughout without abnormal movements or pronator drift. Strength testing revealed symmetric 4/5 strength in both upper and lower extremities; however, examination was limited by patient effort and generalized fatigue, with giveaway weakness noted. Sensory examination demonstrated mildly decreased light-touch sensation in the left upper extremity, while sensation in the lower extremities was intact. Cerebellar testing showed no dysmetria or ataxia on finger-to-nose or heel-to-shin testing. Deep tendon reflexes were 2+ and symmetric at the biceps, triceps, brachioradialis, patellar, and Achilles tendons bilaterally. Gait assessment was deferred because the patient was unable to ambulate secondary to limited effort. The patient had a National Institutes of Health Stroke Scale (NIHSS) score of 1.

Stroke neurology was consulted after initial CT of the head demonstrated age-advanced microvascular ischemic changes versus possible PRES, as well as a punctate hyperdensity within the right parietal deep white matter suggestive of a small hemorrhage or calcification.

During hospitalization, the patient remained intermittently febrile and continued to report headaches, dysarthria, tremors, myalgias, photophobia, and progressive weakness requiring wheelchair assistance for ambulation. Brain MRI performed on hospital day 3 demonstrated patchy bilateral white matter fluid-attenuated inversion recovery (FLAIR) hyperintensities involving the parietal lobes greater than the frontal lobes, as shown in Figure 1, and corresponding diffusion-weighted imaging, as shown in Figure 2. Evaluation was limited by motion artifact, and the findings were considered nonspecific. The radiologic differential diagnosis included migraine-related white matter changes, age-advanced microvascular ischemic disease, inflammatory or autoimmune disorders, central nervous system vasculitis, post-infectious inflammatory syndromes, and demyelinating disease. Demyelination was initially considered after cervical spine MRI demonstrated several questionable T2 hyperintense foci within the cervical cord; however, repeat high-resolution cervical MRI performed on hospital day 5 demonstrated normal cord signal without discrete T2 lesions or abnormal enhancement, suggesting that the previously identified abnormalities were artifactual. The absence of definitive spinal cord lesions or enhancing abnormalities made an active demyelinating process less likely.

**Figure 1:**
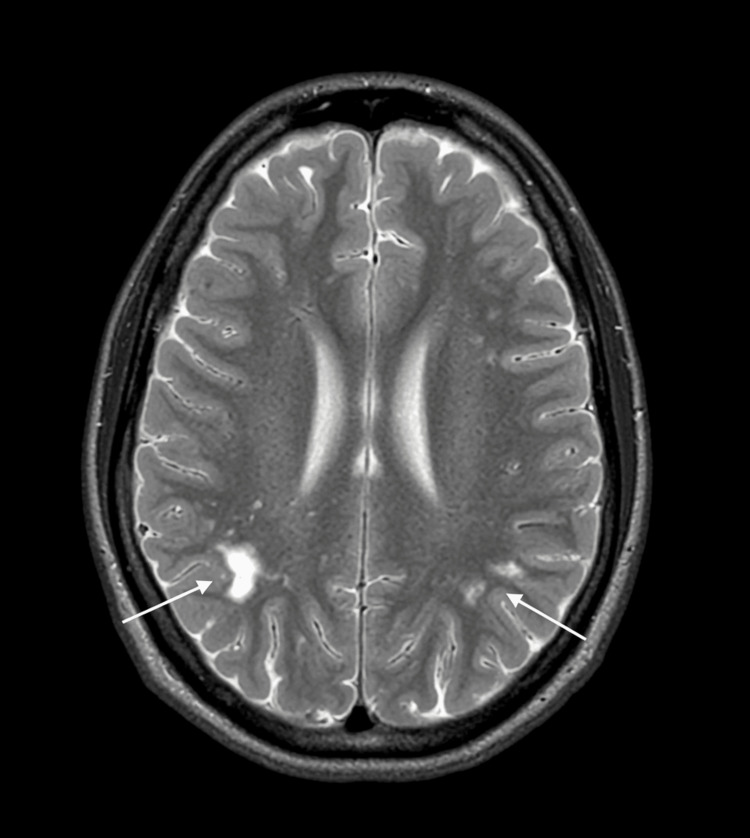
MRI brain (FLAIR) Patchy bilateral white matter FLAIR signal abnormalities (arrows) involving the bilateral parietal greater than the frontal lobes are nonspecific for etiology and could represent sequela of migraine headaches, age-advanced microvascular ischemic changes in the appropriate clinical setting, or, less likely, demyelination, among other potential etiologies. FLAIR: fluid-attenuated inversion recovery

**Figure 2:**
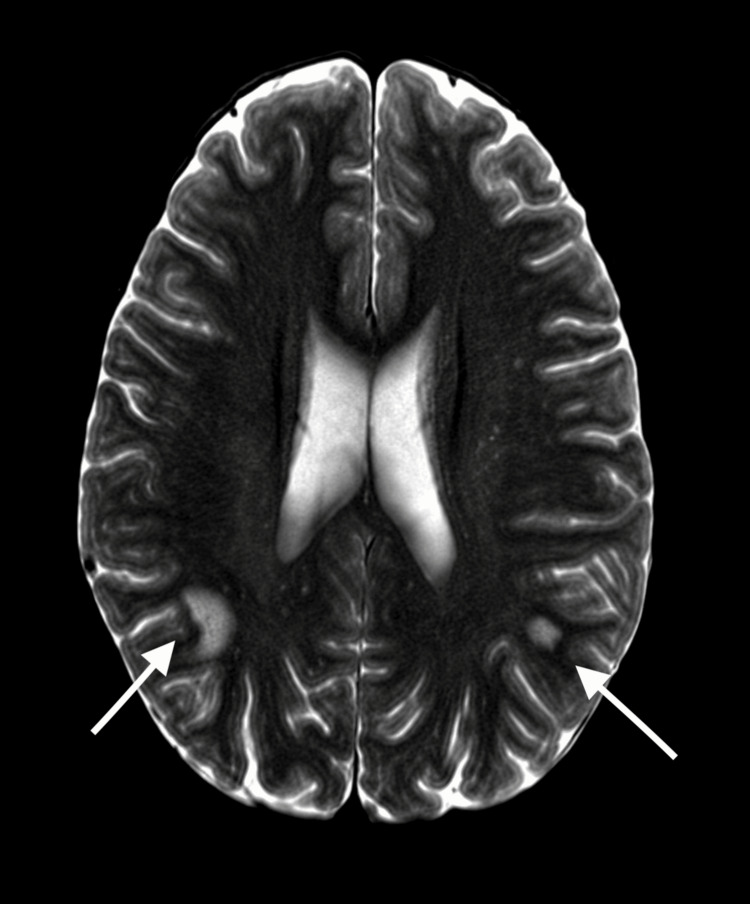
MRI brain (DWI) Patchy bilateral hyperintensities in the bilateral parietal lobes greater than the frontal lobes are nonspecific for etiology and could represent sequela of migraine headaches, age-advanced microvascular ischemic changes in the appropriate clinical setting, or, less likely, demyelination, among other potential etiologies. DWI: diffusion-weighted imaging

The punctate right parietal hyperdensity identified on the initial CT scan was not associated with evidence of intraparenchymal hemorrhage on subsequent MRI, and no corresponding enhancing lesion was identified. As no repeat brain imaging was performed, the precise nature of this finding could not be definitively established and may have represented a small calcification, artifact, or clinically insignificant lesion. Despite the nonspecific imaging appearance, the combination of acute febrile illness, neurologic symptoms, and parietal-predominant white matter abnormalities raised concern for possible PRES-like changes; however, the absence of follow-up imaging precluded confirmation of radiographic reversibility.

Lumbar puncture revealed a normal total nucleated cell count, normal glucose, and low protein. Cerebrospinal fluid (CSF) studies, including a meningoencephalitis polymerase chain reaction (PCR) panel, bacterial cultures, fungal cultures, and acid-fast bacilli cultures, were negative (Table 1). Additional infectious and autoimmune evaluation was unrevealing, including a negative respiratory viral panel, syphilis serology, HIV screening, and autoimmune antibodies (Table 2). Inflammatory markers were elevated with a C-reactive protein of 61 mg/L (reference: <10 mg/L) and an erythrocyte sedimentation rate of 50 mm/hr (reference: 2-37 mm/hr), as seen with other admission laboratory values (Table 3). Blood cultures remained negative.

**Table 1:** CSF counts, meningitis/encephalitis panel, and autoimmune CSF results

Tests	Result	Reference
CSF Tests		
Total Nucleated Cells	<5, <5 /mcL	0
RBCs	1, 0 /mcL	0
Lymphocytes	85%, 87%	40%-80%
Monocytes	7%, 12%	15%-45%
Neutrophils	7%, 1%	0%-6%
Glucose	75 mg/dL	40 - 75 mg/dL
Albumin	6 mg/dL	0 - 35 mg/dL
Protein	11 mg/dL (L)	15 - 45 mg/dL
Oligoclonal Bands	Negative	Negative
Meningitis/Encephalitis Panel		
Cryptococcus neoformans/gattii	Not Detected	Not Detected
Cryptococcus Ag	Not Detected	Not Detected
Cytomegalovirus	Not Detected	Not Detected
Enterovirus	Not Detected	Not Detected
Escherichia coli K1	Not Detected	Not Detected
Herpes Simplex Virus 1	Not Detected	Not Detected
Herpes Simplex Virus 2	Not Detected	Not Detected
Human herpesvirus 6	Not Detected	Not Detected
Human parechovirus	Not Detected	Not Detected
Streptococcus agalactiae (Group B)	Not Detected	Not Detected
Varicella zoster virus	Not Detected	Not Detected
Haemophilus influenzae	Not Detected	Not Detected
Listeria monocytogenes	Not Detected	Not Detected
Neisseria meningitidis	Not Detected	Not Detected
VDRL (Venereal Disease Research Laboratory)	Not Detected	Not Detected
Streptococcus pneumoniae	Not Detected	Not Detected
Autoimmune Labs		
NMO/Aquaporin-4-IgG FACS	Not Detected	Not Detected

**Table 2:** Serum infectious/autoimmune/hepatology labs

Test	Result	Reference
CMV IgM	<0.2	<0.8
Bartonella Henselae Antibody IgG	<1:64	<1:64
Bartonella Henselae Antibody IgM	<1:16	<1:16
Coxiella bunetii Antibody IgG Phase 1	Negative	Negative
Coxiella bunetii Antibody IgG Phase 2	Negative	Negative
Rickettsia Typhi Antibody IgG	<1:64	<1:64
Rickettsia Typhi Antibody IgM	1:1024	<1:64
HIV-1 Quantitative	<20 copies/mL	<20 copies/mL
Quantiferon TB Gold Plus,	Negative	Negative
West Nile Virus Antibody IgG	0.61	<1.29
West Nile Virus Antibody IgM	0.02	<0.89
JC Virus Antibody	Negative	Negative
Hepatitis B Surface Antibody	23 mIU/mL	>10 mIU/mL
Hepatitis B Core Antibody	Non-Reactive	Non-Reactive
Hepatitis B Surface Antigen	Non-Reactive	Non-Reactive
Hepatitis C Antibody	Non-Reactive	Non-Reactive

**Table 3:** Labs on admission

Lab on Admission	Result	Reference
Chemistry Profile		
Sodium	139 mmol/L	135 - 145 mmol/L
Potassium	4.8 mmol/L (slight hemolysis)	3.5 - 5.1 mmol/L
Chloride	106 mmol/L	94 - 106 mmol/L
Carbon Dioxide	24 mmol/L	20 - 29 mmol/L
Urea Nitrogen, Blood	7 mg/dL	7 - 25 mg/dL
Creatinine	1.26 mg/dL (baseline 0.9-1.0) (H)	0.50 - 1.10 mg/dL
Glomerular Filtration Rate Estimated	59 mL/min/1.73m²	>60 mL/min/1.73m²
Alkaline Phosphatase	43 U/L (L)	45 - 117 U/L
Albumin	3.1 g/dL (L)	3.2 - 5.0 g/dL
Total Protein	6.7 g/dL	6.2 - 8.1 g/dL
Lipase	41 U/L	12 - 53 U/L
Aspartate Aminotransferase, AST	64 U/L (H)	<32 U/L
Alanine Aminotransferase, ALT	46 U/L(H)	<36 U/L
Bilirubin, Total	0.38 mg/dL	0.3 - 1.2 mg/dL
Complete Blood Count		
WBC	4.54 K/mcL	3.40 - 10.40 K/mcL
Red Blood Cell Count	4.25 M/mcL	4.00 - 5.00 M/mcL
Hemoglobin	12.9 g/dL	11.5 - 14.9 g/dL
Hematocrit	38	36.0 - 45.5 %
MCV	89.40%	77.7 - 93.7 fL
Platelets	275 K/mcL	140 - 377 K/mcL
Miscellaneous Serum Labs		
Creatinine Kinase	152 U/L (H)	34 - 145 U/L
Sedimentation Rate	50 mm/hr (H)	2 - 37 mm/hr
C-Reactive Protein	61 mg/L (H)	<10.00 mg/L
Urinalysis		
Color, Clarity	Yellow, Clear	Yellow, Clear
pH	5.5	4.5-8
Specific Gravity	1.025	>1.005 - <1.030
Protein, Urine	Negative	Negative
Glucose, Urine	Negative	Negative
Bilirubin, Urine	Negative	Negative
Blood, Urine	Negative	Negative
Nitrites, Urine	Negative	Negative
Leukocytes, Urine	Negative	Negative

The patient's prolonged febrile illness, accompanied by weight loss, night sweats, and early satiety, prompted consideration of a broad differential diagnosis, including occult infection, systemic inflammatory disease, granulomatous disorders, demyelinating disease, and hematologic or solid-organ malignancy. Extensive evaluation was unrevealing. Blood cultures remained negative, CSF studies showed no evidence of bacterial, fungal, mycobacterial, or viral central nervous system infection, and serologic testing for HIV, syphilis, tuberculosis, West Nile virus, Bartonella, Coxiella, and other infectious etiologies was negative. Autoimmune evaluation was similarly unrevealing. No clinical, laboratory, physical examination, or radiographic findings were identified to support an alternative inflammatory, granulomatous, demyelinating, or neoplastic process.

Given persistent fevers and neurologic symptoms with unrevealing diagnostic testing, an infectious disease specialist was consulted. Empiric treatment with doxycycline 100 mg twice daily was initiated for possible atypical infection.

Within 48 hours of starting doxycycline, the patient experienced marked clinical improvement with resolution of fever, dysarthria, tremors, photophobia, and severe headache. Strength gradually improved, and she was able to ambulate again. She was discharged to complete a 7-day course of doxycycline.

Post-discharge serologic testing demonstrated a positive *Rickettsia typhi* IgM titer of 1:1024 with a negative IgG (<1:64). While these findings were consistent with possible early murine typhus in the appropriate clinical context, the absence of convalescent serologic testing limited definitive confirmation. The patient was contacted after discharge and scheduled for follow-up evaluation with repeat serologic testing; however, she did not attend the appointment because of financial constraints, lack of health insurance, and difficulty finding someone to watch her children. Despite the absence of follow-up testing, she reported significant improvement in symptoms after completing doxycycline therapy. The complete chronological timeline of the patient's clinical course, from initial symptom onset through post-discharge follow-up, is illustrated in Figure 3

**Figure 3:**
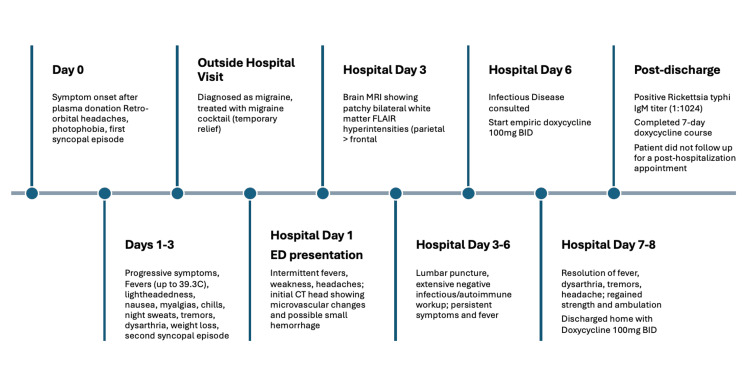
Chronological timeline of clinical presentation, diagnostic evaluations, and treatment course from symptom onset (Day 0) through post-discharge follow-up

## Discussion

Epidemiology and pathogenesis of murine typhus

Murine typhus, also known as endemic or flea-borne typhus, is an acute febrile zoonosis caused by the obligately intracellular Gram-negative coccobacillus *Rickettsia typhi* [5,6]. It is classically characterized by the triad of fever, headache, and rash; however, the diagnosis is often missed because of its nonspecific presentation. Although many infections are self-limited, delayed recognition and lack of timely antibiotic therapy can result in severe complications, including multiorgan dysfunction and neurologic sequelae [5,7].

Murine typhus has a global but focal epidemiologic distribution. In the United States, cases occur predominantly in Texas, California, and Hawaii [7]. Widespread pest control measures led to a significant decline in incidence during the mid-20th century. The disease has reemerged in suburban areas, especially in Southern California and South Texas [5,8].

Transmission of *Rickettsia typhi *occurs through two main ecological cycles: urban and suburban [5,7]. In the urban cycle, the Oriental rat flea (Xenopsylla cheopis) is the primary vector. Roof rats (Rattus rattus) and Norway rats (Rattus norvegicus) are the main reservoirs [8,9]. The suburban cycle, responsible for most modern US cases, involves the cat flea (Ctenocephalides felis) as the primary vector. Opossums (Didelphis virginiana) and free-roaming cats serve as the main reservoirs [5,8]. Humans are typically infected not by flea bites, but by inoculation of infected flea feces into bite sites, abrasions, or mucous membranes via scratching or contact [5,6].

Neurologic manifestations of murine typhus

Although murine typhus is frequently described as a relatively mild febrile illness, neurologic involvement has been reported and may contribute substantially to morbidity. *Rickettsia typhi *exhibits a predilection for vascular endothelial cells, producing a systemic vasculopathy that is thought to underlie many of the disease's complications. In cases with central nervous system involvement, endothelial injury and disruption of the blood-brain barrier may contribute to inflammatory neurologic manifestations, including meningitis and encephalitis [5]. Neurologic manifestations are uncommon but well-documented, occurring in approximately 2-5% of reported cases from endemic regions [3].

Aseptic meningitis is among the most commonly reported neurologic complications of murine typhus and typically presents with severe headache, photophobia, and nuchal rigidity. In such cases, CSF analysis often demonstrates lymphocytic pleocytosis with mildly elevated protein levels [1]. Less commonly, patients may develop encephalitis characterized by altered mental status, seizures, or focal neurologic deficits. Neuroimaging findings in patients with neurologic involvement are generally nonspecific and may include cerebral edema, white-matter abnormalities, or findings suggestive of vasculitic injury [5]. In contrast, our patient demonstrated normal CSF studies and posterior-predominant white-matter abnormalities interpreted as concerning for PRES. Because neither acute murine typhus nor PRES was definitively confirmed, these findings should be interpreted cautiously. Nonetheless, the case illustrates the diagnostic complexity of febrile illnesses with neurologic manifestations and highlights the challenges of attributing atypical neuroimaging findings to a specific infectious etiology.

The neurologic manifestations of murine typhus may overlap with those of other infectious, inflammatory, and vascular disorders, creating significant diagnostic challenges. Consequently, the diagnosis may be delayed or overlooked, particularly when classic clinical features are absent. Although doxycycline is typically effective, some patients with neurologic involvement may experience persistent sequelae, including cognitive impairment or sensorineural hearing loss [5]. In the present case, the combination of febrile illness, neurologic symptoms, isolated *Rickettsia typhi *IgM positivity, and posterior white-matter abnormalities raises the possibility of an association with murine typhus; however, given the absence of definitive confirmation of either acute murine typhus or PRES, the relationship should be interpreted cautiously.

PRES-like neuroimaging in murine typhus

Several aspects of this case distinguish it from typical presentations of murine typhus. First, the patient demonstrated neuroimaging findings concerning for possible PRES-like features. PRES is a clinico-radiologic syndrome characterized by vasogenic edema predominantly affecting the parietal and occipital lobes and is most commonly associated with severe hypertension, renal failure, eclampsia, immunosuppressive medications, or systemic inflammatory states [10]. In this case, MRI demonstrated patchy bilateral white matter FLAIR signal abnormalities involving the parietal lobes more prominently than the frontal lobes, as seen in Figure 1. Infectious etiologies have increasingly been recognized as potential triggers of PRES through mechanisms involving endothelial dysfunction, blood-brain barrier disruption, and dysregulated cerebral autoregulation. Given the known endothelial tropism of *Rickettsia typhi*, it is biologically plausible that rickettsial infection could contribute to the development of vasogenic edema and PRES-like imaging abnormalities [11]. However, to our knowledge, PRES has not been definitively reported in association with murine typhus. Therefore, the relationship between the patient's febrile illness, isolated *Rickettsia typhi* IgM positivity, and posterior white-matter abnormalities remains speculative. While this case raises the possibility of an association, it does not establish a causal link between murine typhus and PRES-like neuroimaging findings.

An important limitation of this case is the absence of follow-up neuroimaging. Repeat MRI was not obtained after clinical recovery; therefore, resolution of the white matter abnormalities could not be confirmed. As radiographic reversibility is a defining feature of PRES, the diagnosis of PRES cannot be definitively established in this patient. Consequently, the imaging findings are more appropriately described as posterior-predominant white matter abnormalities with possible PRES-like features rather than confirmed PRES.

Neuroimaging findings in murine typhus with neurologic involvement

Neuroimaging abnormalities in murine typhus are infrequently reported and typically nonspecific, reflecting the underlying endothelial injury and vasculopathy. A focused literature review reveals only isolated cases with white matter or regional encephalopathic changes, underscoring the unusual nature of PRES-like parietal-predominant FLAIR hyperintensities in our patient [5]. 

One report described a patient with severe murine typhus who developed seizures and coma. Brain MRI demonstrated extensive T2 hyperintensities and cortical swelling involving the bilateral temporal and parietal lobes (with medial temporal/hippocampal extension), consistent with diffuse encephalitis. Follow-up imaging showed bitemporal encephalomalacia and atrophy. This case shares parietal involvement and neurologic sequelae but featured more prominent temporal changes and a fulminant course [12].

Another case presented with multiple diffuse cerebral white matter lesions and infarctions on MRI, highlighting that rickettsial vasculopathy can produce multifocal white matter abnormalities mimicking other inflammatory or demyelinating processes [13].

Additional reports document cerebral edema, petechial white matter changes, or watershed ischemic necrosis, but detailed antemortem MRI descriptions remain scarce. In contrast to more commonly associated rickettsioses (e.g., scrub typhus or spotted fever group infections, where PRES has been documented in rare cases linked to endothelial dysfunction), no prior reports to our knowledge describe PRES-like posterior/parietal vasogenic edema in confirmed murine typhus [5,14].

While these reports demonstrate that neurologic involvement and neuroimaging abnormalities can occur in murine typhus, the findings in our patient remain atypical. The combination of PRES-like imaging abnormalities, normal CSF studies, rapid response to doxycycline, and isolated *Rickettsia typhi* IgM positivity has not been widely described and should be interpreted cautiously.

Diagnostic challenges

Second, severe neurologic manifestations of murine typhus have been reported in older adults and in patients with underlying medical conditions, including diabetes, chronic kidney disease, and immunosuppression [15]. However, neurologic involvement can also occur in otherwise healthy individuals. The occurrence of significant neurologic symptoms in this young immunocompetent patient highlights the importance of considering rickettsial infections in the differential diagnosis of unexplained febrile illnesses with neurologic manifestations. Importantly, the patient's residence in Texas, where murine typhus remains endemic, provided an epidemiologic clue that increased clinical suspicion despite the absence of a recognized flea or animal exposure.

Third, the patient’s CSF findings were atypical. While rickettsial infections involving the CNS often demonstrate mild lymphocytic pleocytosis and elevated protein levels, CSF studies may occasionally be normal or only minimally abnormal [16]. In this case, CSF analysis demonstrated normal nucleated cell count and glucose with low protein, and extensive infectious and autoimmune testing was unrevealing. These findings initially broadened the differential diagnosis to include demyelinating disease, migraine-related changes, and other inflammatory etiologies, illustrating how normal CSF results do not exclude rickettsial infection.

An additional diagnostic consideration was the presence of age-advanced white matter abnormalities on initial neuroimaging. Such findings are unusual in a previously healthy woman in her 30s and prompted evaluation for alternative vascular, inflammatory, autoimmune, and demyelinating processes. However, extensive laboratory, cerebrospinal fluid, and imaging studies did not identify evidence of systemic vasculitis, autoimmune disease, active demyelination, or another underlying disorder to explain the radiographic findings. Although the significance of these white matter abnormalities remains uncertain, their appearance in the setting of an acute febrile illness, coupled with the absence of evidence supporting an alternative chronic neurologic process, raised the possibility that they were related to the patient's acute presentation.

Role of empiric doxycycline

Another notable feature was the patient’s rapid clinical improvement following empiric doxycycline therapy. Doxycycline remains the treatment of choice for rickettsial infections and is associated with rapid defervescence and symptomatic improvement, often within 24-48 hours [17]. Definitive serologic testing for flea-borne murine typhus may take several days to weeks, with early serologies often negative due to delayed antibody production. For this reason, clinical guidelines emphasize initiating empiric doxycycline when rickettsial infection is suspected rather than delaying treatment while awaiting confirmatory testing [17]. In this case, the patient’s rapid improvement following the initiation of doxycycline served as both a therapeutic intervention and an important diagnostic clue.

## Conclusions

Overall, this case highlights the diagnostic challenges of a febrile illness with neurologic manifestations and MRI abnormalities occurring in the setting of suspected murine typhus. While neither acute murine typhus nor possible PRES-like neuroimaging changes could be definitively established, and repeat MRI was not available to demonstrate radiographic resolution, the clinical presentation underscores the importance of considering rickettsial infections in patients with unexplained febrile neurologic syndromes, particularly in endemic areas. Prompt recognition and empiric doxycycline therapy remain important when clinical suspicion is high, as delayed treatment may increase morbidity.
